# Arabidopsis Plasma Membrane ATPase AHA5 Is Negatively Involved in PAMP-Triggered Immunity

**DOI:** 10.3390/ijms23073857

**Published:** 2022-03-31

**Authors:** Zhenzhen Zhao, Jiangbo Fan, Yu G. Gao, Zonghua Wang, Piao Yang, Yinping Liang, Stephen Opiyo, Ye Xia

**Affiliations:** 1 Department of Plant Pathology, College of Food, Agricultural, and Environmental Science, The Ohio State University, Columbus, OH 43210, USA; zhao.2047@osu.edu (Z.Z.); yang.4636@osu.edu (P.Y.); liangyinping@sxau.edu.cn (Y.L.); opiyo.1@osu.edu (S.O.); 2 School of Agriculture and Biology, Shanghai Jiao Tong University, 800 Dongchuan Rd., Shanghai 200240, China; fan.0127@yahoo.com; 3 OSU South Centers, The Ohio State University, 1864 Shyville Road, Piketon, OH 45661, USA; gao.2@osu.edu; 4 State Key Laboratory of Ecological Pest Control for Fujian and Taiwan Crops, Fujian Agriculture and Forestry University, Fuzhou 350002, China; wangzh@fafu.edu.cn; 5 School of Grass Industry, Shanxi Agricultural University, Jinzhong 030801, China

**Keywords:** PM H^+^-ATPases, AHA5, PAMP-triggered immunity, defense responses, stomatal regulation, H_2_O_2_

## Abstract

Plants evolve a prompt and robust immune system to defend themselves against pathogen infections. Pathogen-associated molecular pattern (PAMP)-triggered immunity (PTI) is the first battle layer activated upon the PAMP’s perception, which leads to multiple defense responses. The plasma membrane (PM) H^+^-ATPases are the primary ion pumps to create and maintain the cellular membrane potential that is critical for various essential biological processes, including plant growth, development, and defense. This study discovered that the PM H^+^-ATPase AHA5 is negatively involved in Arabidopsis PTI against the virulent pathogen *Pseudomonas syringae* pvr. *tomato* (*Pto*) DC3000 infection. The *aha5* mutant plants caused the reduced stomata opening upon the *Pto* infection, which was associated with the salicylic acid (SA) pathway. In addition, the *aha5* mutant plants caused the increased levels of callose deposition, defense-related gene expression, and SA accumulation. Our results also indicate that the PM H^+^-ATPase activity of AHA5 probably mediates the coupling of H_2_O_2_ generation and the apoplast alkalization in PTI responses. Moreover, AHA5 was found to interact with a vital defense regulator, RPM1-interacting protein 4 (RIN4), in vitro and in vivo, which might also be critical for its function in PTI. In summary, our studies show that AHA5 functions as a novel and critical component that is negatively involved in PTI by coordinating different defense responses during the Arabidopsis–*Pto* DC3000 interaction.

## 1. Introduction

It is known that plants evolve a sophisticated two-layer defense system, which includes the pathogen-associated molecular pattern (PAMP)-triggered immunity (PTI) and effector-triggered immunity (ETI). PAMPs are conserved pathogen-associated structural components, such as flagellin, lipopolysaccharide (LPS), chitin, peptidoglycan (PGN), etc. The PAMPs can be sensed by the pattern recognition receptors (PRRs) on the plasma membrane of plant cells and trigger the PTI, leading to a series of fast responses, such as the Ca^2+^ influx, reactive oxygen species (ROS) burst, alkalization of the apoplast, activation of mitogen-activated protein kinases (MAPKs), hormone production, callose deposition, pathogenesis-related (PR) gene expression, stomatal closure, etc. [1,2,3,4]. In turn, pathogens secrete virulent factors, such as effectors, to subvert the plant defense responses. Some of the effectors can be recognized, directly or indirectly, by resistance (R) proteins from the hosts and trigger much more vigorous immune responses, namely, the effector-triggered immunity (ETI), which usually leads to a hypersensitive response (HR), conferring resistance to the (hemi-)biotrophic pathogen infections [2,3]. Emerging results also show that PTI is an indispensable component of ETI during bacterial infections, which indicates the complex interplay between PTI and ETI [5,6]. RPM1-interacting Protein 4 (RIN4) is an intrinsically disordered protein that is conserved in land plants. RIN4 was reported to function as a plasma membrane platform or scaffold to mediate the formation of PTI and ETI complexes to regulate PTI or ETI responses [7,8,9,10,11,12]. RIN4 is negatively involved in the PTI and ETI signaling pathways in Arabidopsis plants. The mutants and over-expressed lines of *RIN4* displayed the enhanced and reduced defenses against the bacterial pathogen *Pto*, respectively [13]. 

In plants, PM H^+^-ATPase proteins are constituted by two domains, a cytosolic domain containing the catalytic site and a C-terminal region, which is the auto-inhibitory domain of the ATP hydrolase when it is not phosphorylated. PM H^+^-ATPase energetically couples two reactions, the ATP hydrolysis and the transport of H^+^ from the cytosol to the apoplast, which results in the generation of a chemical gradient of H^+^ (ΔpH), and the establishment of an electrical gradient (membrane potential ΔE) [14,15,16,17]. The H^+^ gradient (ΔpH) is the driving force for several essential processes, such as the secondary transport of nutrients, cell elongation, and stomata opening in different plant species [17]. Arabidopsis evolved 11 plasma membrane H^+^-ATPases (PM H^+^-ATPases) to meet their essential roles for plant growth, development, and defense [17,18]. By applying RT-PCR analysis using the specific primers for each *AHA* isoform, it was found that all *AHA* isoforms express in the guard cell protoplasts, and *AHA1/2/5* are the major ones. *AHA5* is predominantly expressed in the guard cells but is not defined in roots. Eight *AHAs* in green leaf tissues (*AHA1/2/3/5/7/8/10/11*), four *AHAs* in mesophyll cell protoplasts (*AHA1/2/10/11*), and eight in roots (*AHA1/2/3/4/7/8/10/11*) were reported in Arabidopsis plants [19]. *AHA1* and *AHA3* were first cloned from Arabidopsis plants and involved in steroid signaling and pollen development [20,21]. In the following years, researchers reported the roles of *AHA1* in stomatal movement regulation; *AHA2* in iron transport, root elongation, and plant defense [9,22,23]; *AHA4* in salt stress [24]; *AHA7* for root hair formation and in response to low-phosphorus stress [23]; and *AHA10* in vacuole development [25]. 

Stomata are an important battlefield where the plants defend themselves against pathogen invasions by controlling their movements (opening and closure). The stomatal movement is regulated by many factors, including the PAMPs, bacterial effectors, plant defense hormones, blue light, etc. [26,27,28]. The stomata will be closed during the plant–pathogen interactions by perceiving PAMPs and plant hormones, such as abscisic acid (ABA) and salicylic acid (SA), as a plant PTI response against the pathogen infections [28]. On the other hand, bacterial pathogens, such as the *Pseudomonas syringae pv. tomato* (*Pto*), can induce the stomatal re-opening for bacterial entry by delivering an effector AvrB and generating a phytotoxin coronatine (COR) [28]. In Arabidopsis, two closely related and functionally redundant H^+^-ATPases, AHA1 and AHA2, are crucial in the stomatal-involved defense against the bacterial pathogen *Pto* [9,29]. The effector AvrB of the *Pto* bacterium manipulates the phosphorylation of RIN4 to activate AHA1 and AHA2 activity, which leads to the re-opening of stomata, facilitating the bacterial entry for infection [28].

In addition to AHA1 and AHA2, AHA5 is another abundant H^+^-ATPase that is highly expressed in the guard cells of Arabidopsis [19]. However, the related function and mechanism of AHA5 in plant immunity are largely unknown. Our studies first discovered that AHA5 was negatively involved in PTI by affecting a series of defense responses. The *aha5* mutant plants led to the increased levels of stomatal closure, callose deposition, apoplastic alkalization, defense-related gene expression, and defense hormone SA accumulation for PTI in Arabidopsis. Besides, AHA5 may function in coupling the proton (H^+^) pumping with H_2_O_2_ production during the PTI. Interestingly, AHA5 could physically interact with RIN4 like AHA1 and AHA2, which indicates that AHA5 may function together with RIN4 in PTI. Therefore, our study discovered that AHA5 is a critical PM H^+^-ATPase that is negatively involved in PTI in Arabidopsis by affecting multiple defense responses. 

## 2. Results

### 2.1. The aha5 Mutants Displayed an Enhanced Resistance against the Pto Pathogens

**Figure 1:**
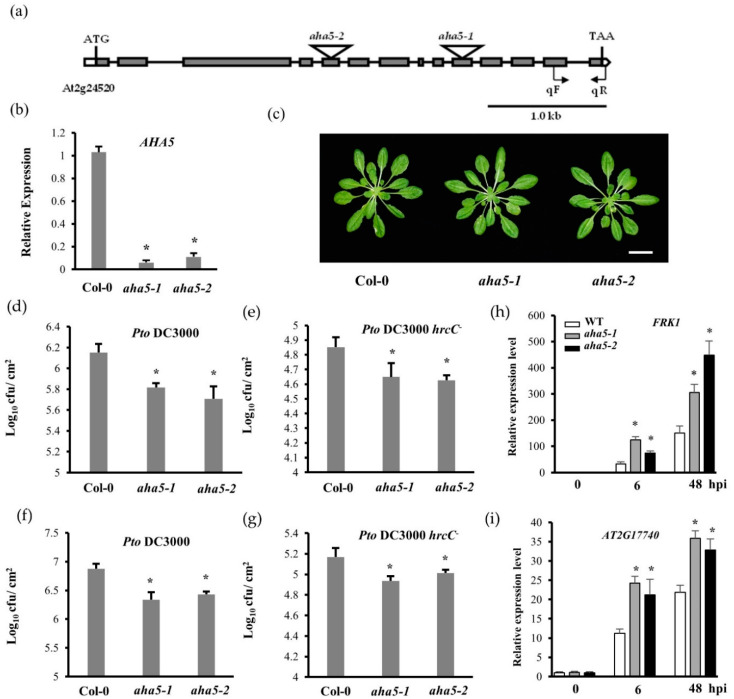
The *aha5* mutants displayed an enhanced resistance against the bacterial pathogen *Pto*. (**a**) The schema of the *AHA5* gene structure and T-DNA insertional sites. Exons and introns are shown in boxes and lines, respectively. The positions and orientation of the primers used for qPCR are labeled as qF and qR. The scale bar indicates 1.0 kb. (**b**) Relative gene expression of *AHA5* in WT Col-0 and *aha5* mutants by qPCR normalized to *ACTIN*’s expression. (**c**) The morphology phenotype of the vegetative rosettes did not differ between 4-week-old *aha5* mutants and WT Col-0 plants. The scale bar indicates 2 cm. (**d**,**e**) The response of WT and *aha5* mutant plants to the *Pto* DC3000 and *Pto* DC3000 *hrcC−* infections by the syringe infiltration of Arabidopsis leaves. The bacterial suspensions of *Pto* DC3000 and *Pto* DC3000 *hrcC*− (at 5 × 10^5^ CFU/mL) were syringe infiltrated into the abaxial side of Arabidopsis leaves. Bacterial populations were quantified at 3 dpi. (**f**,**g**) The response of WT and *aha5* mutant plants to the *Pto* DC3000 and *Pto* DC3000 *hrcC−* infections by spray inoculation of Arabidopsis plants. The bacterial suspension of *Pto* DC3000 and *Pto* DC3000 *hrcC−* (at 1 × 10^9^ CFU/mL) was sprayed onto the leaves. Bacterial populations were quantified at 3 dpi. The syringe infiltration and spray inoculation experiment were repeated three times with similar results. The data represent means ± SE (n = 4) from one of the three independent repeats. Significant differences between WT and *aha5* mutants are indicated by the asterisks and were determined by unpaired two-tailed Student’s *t*-tests (*p* < 0.05). (**h**,**i**) The gene expression of the PTI marker genes *FRK1* and *AT2G17740* in WT Col-0 and *aha5* mutants by qPCR normalized to *UBIQUITIN*’s expression. Expression was shown as the untreated average fold for Col-0. The experiments in (**b**–**i**) were repeated three times with similar results. The data represent means ± SE (n = 4) from one of the three independent repeats. Significant differences between the WT and *aha5* mutants were indicated by asterisks and were determined by unpaired two-tailed Student’s *t*-tests (* *p* < 0.05).

The Arabidopsis genome harbors 11 plasma membrane H^+^ pumps (PM H^+^-ATPases). AHA1, AHA2, and AHA5 had been reported to be the most abundant PM H^+^-ATPases expressed in guard cells [19]. AHA1 and AHA2 are closely related to each other compared to AHA5 based on the phylogenetic analysis and multiple sequence alignment (Figure S1a,b). AHA1 shows 94.5% sequence identity with AHA2 but only 82.1% identity with AHA5. However, AHA5 possesses seven variable regions compared to AHA1/2 (Figure S1b). Therefore, AHA5 may have a divergent role from AHA1 and AHA2. To investigate the biological role of *AHA5* (*At2g24520*), two T-DNA insertional mutant lines were obtained from the Arabidopsis Biological Resources Center (ABRC at The Ohio State University, USA). We arbitrarily named them *aha5-1* (SALK_147597) and *aha5-2* (SALK_127844). These two mutant lines’ T-DNA insertions localized at the 5th and 10th exon, respectively (Figure 1a). These two mutants were identified as homozygous lines by the SALK T-DNA verification protocol designed by the Salk Institute Genomic Analysis Laboratory (http://signal.salk.edu/tdnaprimers.2.html) [30]. Quantitative real-time PCR (qPCR) was carried out to verify the *AHA5* transcript levels of the mutants and wild type (WT) Col-0 plant leaves. It was observed that *AHA5* transcript levels were significantly reduced in the mutants, confirming that they are genuine mutants (Figure 1b). It was observed that the mutant lines of *AHA5* did not show growth defects during the whole growth stages, suggesting *AHA5* might not play a prominent role in Arabidopsis growth and development (Figure 1c and Figure S2). 

To further investigate whether *AHA5* functions in PTI, the *aha5* mutants and WT plants were inoculated with the bacterial pathogen virulent strain *Pto* DC3000 via the syringe infiltration and spray inoculation, respectively. Spray inoculation differs from syringe infiltration by one key point: bacteria penetrate through natural surface openings, such as stomata, by spray inoculation, which tests the host immunity involved with the stomatal function [28,29]. In comparison, syringe infiltration is a way to test the host immunity, ignoring the entry through stomata. The bacterial multiplication was determined three days after the pathogen inoculations. It was found that *aha5* mutants grew fewer bacteria than WT plants by both syringe infiltration and spray inoculation (Figure 1d,f). The enhanced resistance to *Pto* DC3000 *hrcC−* was also observed in *aha5* mutants (Figure 1e,g). *Pto* DC3000 *hrcC−* is a functional type III secretion system mutant strain that is unable to deliver effectors into host cells to suppress PTI [31]. Next, the PTI marker genes, including *FRK1* and *AT2G17740*, were measured in the *aha5* mutants and WT plants against *Pto* DC3000 at the indicated time points. As shown in Figure 1h,i, the transcript levels of *FRK1* and *AT2G17740* did not differ between the *aha5* mutants and WT plants before the inoculation. After the inoculation with *Pto* DC3000, the transcript levels of *FRK1* and *AT2G17740* were induced in both mutants and WT. The transcript levels were significantly higher in *aha5* mutants than WT plants at 6 and 48 h after the inoculation (Figure 1h,i). Based on these results, AHA5 is negatively involved in the PTI of Arabidopsis.

### 2.2. AHA5 Is Involved in the pH Homeostasis of the Cytoplasm and Apoplast during the PTI

**Figure 2:**
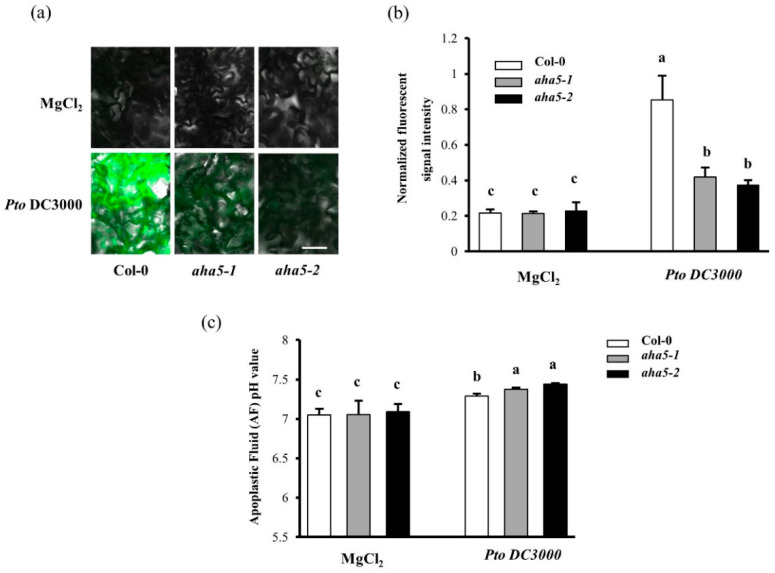
The *aha5* mutant plant cells showed lower pH levels in the cytoplasm and higher pH levels of apoplastic fluid in response to the *Pto* DC3000 treatment. (**a**) The fluorescent image of the cytoplasmic pH alteration in *aha5* mutants and WT upon the *Pto* pathogen infection. The leaves were syringe infiltrated with *Pto* DC3000 (1 × 10^8^ CFU/mL) or 10 mM MgCl_2_. One hour after the treatment, the leaf peel samples were stained with 10 µM BCECF-AM. The fluorescent signal from BCECF-AM was checked under confocal microscopy through the dual-excitation confocal ratio measurement (488 nm/530 nm). The scale bars indicate 50 µm. (**b**) The fluorescent signal intensity in (**a**) was determined with ImageJ software. (**c**) The apoplastic fluid pH alteration in *aha5* mutants and WT upon the *Pto* pathogen infection. The leaves were foliar sprayed with 1 × 10^9^ *Pto* DC3000 suspension or 10 mM MgCl_2_. The apoplastic fluid was extracted 24 h after treatments, and the pH value was measured immediately using a micro-electrode. The data represent means ± SE (n = 4) from one of the three independent repeats with consistent results. Different letters a–c within the figure (**a**–**c**) indicate significant differences at *p* < 0.05, which were calculated by a one-way analysis of variance (ANOVA) using SPSS ver. 21(IBM SPSS Statistics, New York, NY, USA).

Since H^+^-ATPases pump cytoplasmic H^+^ out of plasma membranes, more H^+^-ATPase activity can lead to more significant pH elevation in the cytoplasm [32,33]. It was reported that the cytoplasmic pH could be monitored with the pH-sensitive dye BCECF-AM, which is an intracellular ratiometric pH indicator that displays more green fluorescent signal in the cytoplasm at a higher pH condition [34,35].To investigate the activity and function of *AHA5* in PTI, the plant leaves of *aha5* mutants and WT plants were inoculated with *Pto* DC3000 or MgCl_2_ buffer and then stained with 10 µM BCECF-AM for 15 min. The fluorescent signal of the leaf peel samples was checked under confocal microscopy after a thorough rinsing. Our results showed that the mutants showed the similar low fluorescent intensity as WT when treated with MgCl_2_ (Figure 2a,b). As expected, WT and mutant plants showed more fluorescent signals in response to the *Pto* infection than MgCl_2_ treatment, which indicates that H^+^-ATPase activity was induced upon the *Pto* infection. However, the *aha5* mutants had a significantly weaker fluorescent signal intensity than WT plants after the *Pto* treatment, suggesting that the *aha5* mutants possessed a defective H^+^-ATPase activity compared to WT plants (Figure 2a,b).

On the other hand, the apoplastic fluid pH changes were monitored in *aha5* mutants and WT plant leaves in response to the *Pto* DC3000 infection. When *aha5* mutants and WT plant leaves were foliar sprayed with the *Pto* DC3000 or MgCl_2_ treatment for 24 h, the apoplastic fluid (AF) from the *aha5* mutants and WT plant leaves was extracted by the infiltration-centrifuge protocol method [36]. It was found that the apoplastic fluid pH increased in both mutant and WT plant leaves in response to the *Pto* DC3000 infection. Consistent with the results of the cytoplasmic pH changes, the alkalinization of the apoplast was more apparent in *aha5* mutants than WT plants against *Pto* (Figure 2c). Taken together, *AHA5* plays a vital function in PTI, which might be related to the H^+^-ATPase activity of *AHA5* during the host–pathogen interactions.

### 2.3. AHA5 Is Required for the PTI-Induced Apoplastic H_2_O_2_ Accumulation

**Figure 3:**
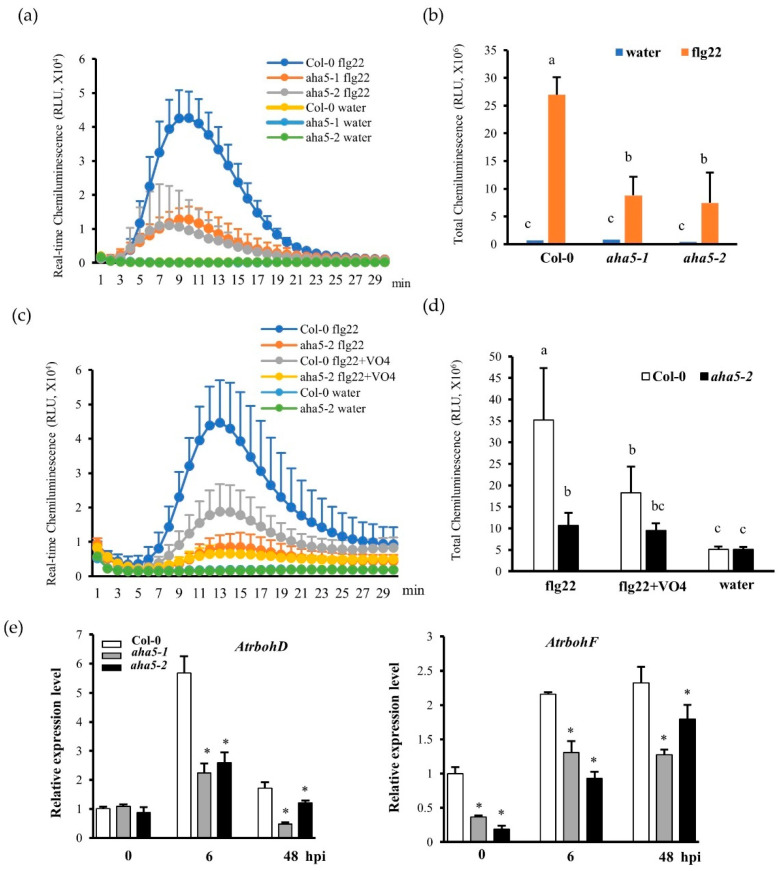
The *aha5* mutant plants showed the decreased H_2_O_2_ production and lower levels of gene expressions related to ROS production in response to the PAMP treatment. (**a**). The real-time H_2_O_2_ burst curve of *aha5* mutants and Col-0 plants in response to the PAMP flg22 and water treatments. Leaf discs (7 mm in diameter) of *aha5* mutants and Col-0 plants were pretreated with sterile water for 18 h. Those pretreated leaf discs were further treated with water and 1 μM flg22. The H_2_O_2_ induction was monitored by checking the luminescence for 30 min using a GLOMAX luminometer. (**b**) The total H_2_O_2_ production analyzed from the real-time H_2_O_2_ burst curve of *aha5* mutants and Col-0 plants upon 1 μM flg22 induction and water treatment. This experiment was repeated three times with similar results. (**c**) The real-time H_2_O_2_ burst curve of *aha5-2* mutant and Col-0 plants to the flg22 induction with and without sodium vanadate (VO4) treatment. The leaf discs of *aha5-2* mutant and Col-0 plants were treated with water or 1 μM flg22 (with and without VO4 treatment). The H_2_O_2_ generation was monitored by checking the luminescence for 30 min using a GLOMAX luminometer. (**d**) The total H_2_O_2_ production of *aha5-2* and Col-0 plants upon 1 μM flg22 induction with and without the VO4 treatment were calculated from the real-time H_2_O_2_ burst curve. The results in (**b**,**d**) represent means ± SE (n = 3), which are from one of the three independent repeats with consistent results. Different letters a–c within the figure (**b**,**d**) indicate significant differences at *p* < 0.05, which were calculated by a one-way analysis of variance (ANOVA) using SPSS ver. 21. (IBM SPSS Statistics, New York, NY, USA). (**e**) The relative gene expression of *AtrbohD* and *AtrbohF* in WT Col-0 and *aha5* mutants was measured by qPCR and normalized to the expression of *UBIQUITIN*. The data represent means ± SE (n = 4) from one of the three independent repeats with consistent results. Expression was shown as the untreated average fold for Col-0. Significant differences between WT and *aha5* mutants were indicated by asterisks and were determined from unpaired two-tailed Student’s *t*-tests (* *p* < 0.05).

Apoplastic ROS accumulation is one of the earliest plant defense responses during PTI [37,38,39]. H^+^-ATPases (AHAs) function as an important ion pump, transporting H^+^ from the cytosol to the apoplast. Thus, the H^+^ transport activity of AHAs might be correlated with ROS accumulation. To further study if *AHA5* is involved in PTI-induced H_2_O_2_ accumulation, the real-time H_2_O_2_ production was monitored in *aha5* mutants and WT plants in response to the PAMP flg22 (a 22 amino acid epitope of flagellin) treatment. The perception of flg22 by its receptor FLAGELLIN SENSITIVE2 (FLS2) normally leads to H_2_O_2_ production. Our results showed that *aha5* mutants produced less H_2_O_2_ than WT Col-0 plants at different time points (Figure 3a,b). To further validate the involvement of *AHA5* in H_2_O_2_ production, the H_2_O_2_ burst assay was carried out with the H^+^-ATPase inhibitor sodium vanadate. With sodium vanadate (VO4) treatment, H^+^-ATPase activities (H^+^ out-pumping activities) were suppressed, and the PAMP-induced H_2_O_2_ production could be reduced [40,41,42]. As expected, the PAMP-flg22-induced H_2_O_2_ production was sharply reduced in WT Col-0 leaf samples with VO4 treatment, which confirmed that the H^+^-ATPase activities (H^+^ out-pumping activities) are involved in H_2_O_2_ production (Figure 3c,d). However, upon the PAMP flg22 induction, the mutant *aha5-2* did not show a significantly reduced level of H_2_O_2_ with the H^+^-ATPase inhibitor VO4 treatment compared to without VO4 treatment (Figure 3c,d), supporting the notion that AHA5 activity (pumping H^+^ from the cytosol and supplying apoplastic H^+^) might be involved in the PTI-induced H_2_O_2_ production. This result is consistent with the lower cytoplastic pH level and higher apoplastic fluid pH level (increased apoplastic alkalinization) in *aha5* mutant plant cells in response to the *Pto* DC3000 treatment, as stated previously (Figure 2a–c). Next, the transcript levels of two critical *RBOHs* for apoplastic ROS accumulation in Arabidopsis, *AtrbohD* and *AtrbohF*, were measured in response to the *Pto* DC3000 infection [43,44]. Consistent with the reduced H_2_O_2_ accumulation in response to PAMP treatment, lower levels of *AtrbohD* and *AtrbohF* transcripts were observed in *aha5* mutants than WT after the pathogen inoculation (Figure 3e). Based on our results, *AHA5* might be required for PTI-induced H_2_O_2_ accumulation, possibly through its H+ out-pumping activity. A summary of the involvement of AHA5 in H_2_O_2_ production is shown in Figure S3.

### 2.4. The aha5 Mutant Plants Showed Enhanced Callose Deposition upon the PAMP Treatment

**Figure 4:**
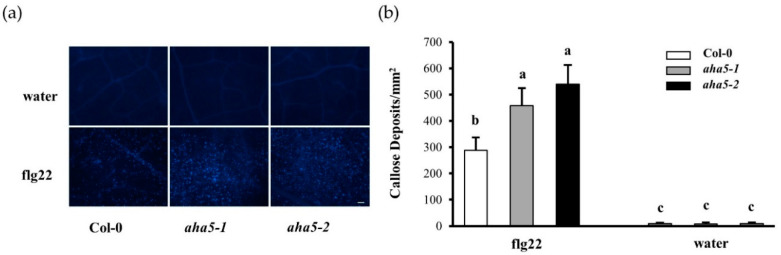
The *aha5* mutant plants increased the callose deposition upon the PAMP flg22 treatment. (**a**) The images of callose deposition upon the PAMP flg22 induction under the microscopy. Four-week-old plants were infiltrated with 50 µM flg22 or water. The leaves were harvested for aniline blue dye staining 16 h after the infiltration. (**b**) Quantification of callose deposition was determined with ImageJ software. The data represent means ± SE (n = 15) from one of the three independent repeats with consistent results. Different letters a–c within the figure (**b**) indicate significant differences at *p* < 0.05, which were calculated by a one-way analysis of variance (ANOVA) using SPSS ver. 21. (IBM SPSS Statistics, New York, NY, USA).

Callose is the reinforcement of the plant cell wall against the pathogen infections. Callose deposition is another hallmark of the early PTI defense response in addition to ROS accumulation. To further study the involvement of *AHA5* in this aspect of PTI, the callose deposition assay was conducted in *aha5* mutants and WT with flg22 treatment. The infiltrated leaves were cut and stained with aniline blue dye solution 14 h after the flg22 treatment. The results showed that *aha5* mutants accumulated more callose than WT Col-0 plants in response to PAMP treatment (Figure 4a,b). Col-0 plants produced around 287.3 (±49.5) while *aha5-1* and *aha5-2* plants produced 457.2 (±49.5) and 539.2 (±49.5) callose dots per mm^2^. Water treatment was carried out as a negative control, which did not induce callose deposition on Col-0 and *aha5* mutant leaves. Altogether, *AHA5* was involved in PTI responses by affecting the callose deposition.

### 2.5. The aha5 Mutant Plants Affected the Stomata Apertures in the Pathogen and Hormone Treatments

**Figure 5:**
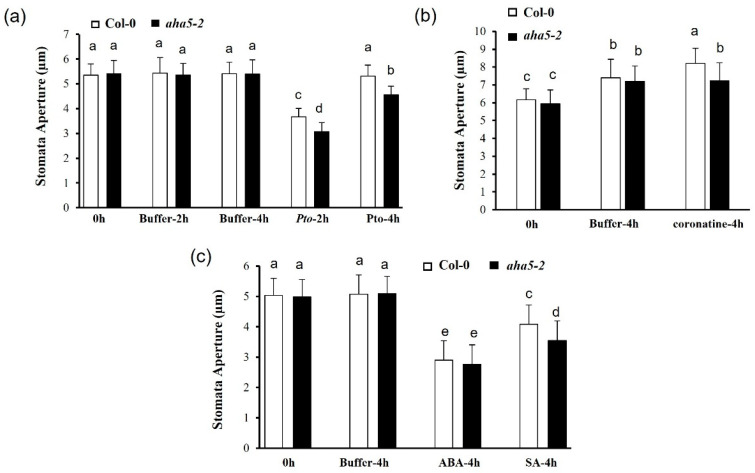
*AHA5* is involved in the stomatal movement upon the pathogen and hormone treatments. (**a**) The *aha5-2* mutant showed the enhanced stomatal closure against the *Pto* pathogen. At the six-week-old stage, the *aha5-2* mutant and WT Col-0 leaves were treated with the cell suspension (10^8^ CFU/mL) of the virulent strain *Pto* DC3000. The stomatal apertures were checked with microscopy at 0 h, 2 h, and 4 h after the pathogen infection with the abaxial epidermis peels. (**b**) The *aha5-2* mutant plants did not respond to the coronatine treatment as the WT plants did. The *aha5-2* mutant and WT Col-0 leaves at the six-week-old stage were placed in the buffer or buffer containing 1 ng/µL coronatine. Stomatal apertures were checked with microscopy at 0 h and 4 h after the treatments. (**c**) The *aha5-2* mutant plants displayed the SA-induced stomatal closure compared to the WT plants. The *aha5-2* mutant and WT Col-0 leaves at the six-week-old stage were treated with 50 μM ABA, 500 μM SA, or MES buffer. The stomatal apertures were checked at 0 h and 4 h after the treatments. All the experiments related to stomata regulation with different treatments were repeated three times with similar results. The data represent means ± SE (n = 50), which are from one of the three independent repeats. Different letters a–e within the figures (**a**–**c**) indicate significant differences at *p* < 0.05, which was calculated by a one-way analysis of variance (ANOVA) using SPSS ver. 21. (IBM SPSS Statistics, New York, NY, USA).

PM H^+^-ATPases 1 and 2 (AHA1 and AHA2) had been well studied for their functions in plant immunity through regulating stomatal movements and related function during bacterial attacks [9]. To investigate whether *AHA5* is involved in stomata-involved defense, the stomata movement assays were conducted to determine the stomatal responses to the bacterial pathogen infections and different phytohormone treatments in *aha5* mutant and WT plants. Based on the previous studies, the Arabidopsis plants would activate the defense mechanism to close the stomata to prevent the entry of the *Pto* pathogen in the first 1–2 h. Then, the *Pto* pathogen could manipulate stomata re-opening to exert its virulence at around 4 h post-inoculation. In our study, leaves of *aha5-2* and WT plants were cut off and treated with the cell suspensions of the virulent strain *Pto* DC3000. The stomata apertures were checked at 0 h, 2 h, and 4 h after the *Pto* treatment. Buffer MgCl_2_ treatment was used as a control. The results indicated that *Pto* induced stomatal closure on both WT and *aha5-2* mutant plants at 2 h after the *Pto* treatment. At the time point of 4h, the stomata re-opened in both the Col-0 and *aha5-2* plants. However, *aha5-2* showed significantly smaller stomatal apertures compared to WT plants (Figure 5a). The results indicated that the *aha5-2* mutant could partially prevent the stomata re-opening initiated by the *Pto* pathogen. *Pto* DC3000 is known to generate a critical virulence factor coronatine, an analog of jasmonic acid (JA), to actively re-open the stomata after the PTI-induced stomata closure [45]. Here, the stomata responsiveness to coronatine treatment was analyzed in *aha5-2* mutant and WT plants. The whole plant leaves of *aha5-2* mutant and WT plants were placed in MES buffer and buffer containing 1 ng/µL coronatine, respectively. Stomatal apertures were checked with microscopy at 0 h and 4 h after the treatments. It was found that WT Col-0 plant leaves responded to coronatine with a significantly wider stomatal aperture than *aha5-2* mutant leaves at 4h after treatment (Figure 5b). The control buffer treatment did not show a difference in stomatal apertures between WT and *aha5-2* mutant. This result indicates that the *aha5-2* mutant caused a decreased response to coronatine treatment, suggesting that AHA5 might be involved in the coronatine-mediated stomatal opening.

Plant hormones are also known as vital signals to regulate stomatal development and movements [46,47]. For instance, salicylic acid (SA) and abscisic acid (ABA) had been reported to be involved in stomatal closure and the related defense against the *Pto* infection [28,48]. The stomata assays were conducted to determine if SA and ABA could be involved in the *AHA5*-involved stomatal movement. The results showed that *aha5-2* leaves responded to SA more substantially than WT Col-0, with more significant stomatal closure in the *aha5-2* mutant than WT plants at 4 h after the SA treatment (Figure 5c). However, the *aha5-2* mutant displayed similar responses on stomatal closure as WT plants in response to the ABA treatment (Figure 5c). These results indicated that the *aha5-2* mutant was more sensitive to SA-mediated stomatal closure but not to ABA. The same responses of stomatal movement to the pathogen infection and hormone treatments were also observed in *aha5-1* mutants, which is shown in Figure S4. Taken together, the defective function of *AHA5* leads to the impaired stomatal re-opening in response to *Pto*, which might contribute to the enhanced resistance against the *Pto* pathogens (*Pto* DC3000 and *Pto* DC3000 *hrcC−*) via the spray inoculation. Moreover, the involvement of *AHA5* in stomatal defense might be related to SA but not ABA.

### 2.6. The aha5 Mutants Accumulate Higher Levels of SA and Induce SA-Responsive Defense Genes in Response to the Pto DC3000 Infection

**Figure 6:**
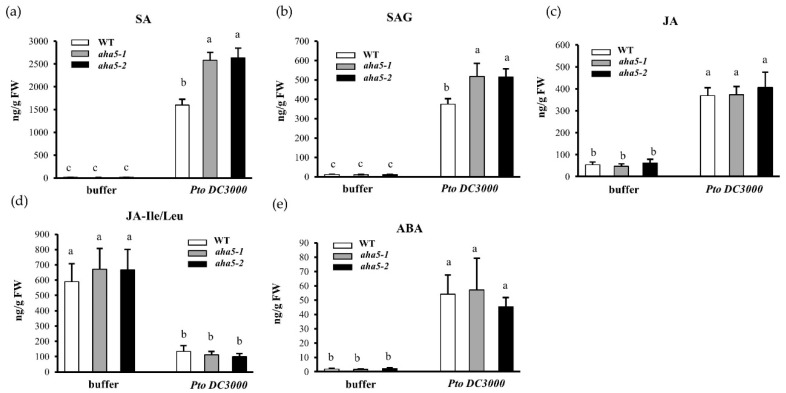
Quantification of hormone levels in *aha5* mutants and WT plants in response to the *Pto* DC3000 infection. The 4-week-old *aha5* mutants and WT plant leaves were syringe infiltrated with *Pto* DC3000 (at 5 × 10^5^ CFU/mL) and buffer MgCl_2_. The leaf samples that were collected for hormone quantification at 48 hpi. (**a**–**e**) show the hormone levels of SA, SAG, JA, JA-Leu/Ile, and ABA in *aha5* mutants and WT plants after *Pto* DC3000 infection. The data represent means ± SE (n = 4) from one of the three independent repeats with consistent results. Different letters a–c within the figures (**a**–**e**) indicate significant differences at *p* < 0.05, which were calculated by a one-way analysis of variance (ANOVA) using SPSS ver. 21. (IBM SPSS Statistics, New York, NY, USA).

To further investigate whether the defense hormone accumulation is affected by the defective function of AHA5 in Arabidopsis plants upon the *Pto* DC3000 infection, the levels of free SA, SA-glycoside (SAG), free JA, JA-isoleucine/leucine (JA-Leu/Ile), and ABA were quantified in *aha5* mutants and WT Col-0 plants at 48 h after the *Pto* DC3000 infections with buffer MgCl_2_ infiltration as a control. As shown in Figure 6, SA and SAG levels increased in both *aha5* mutants and WT plants after the *Pto* infection. However, *aha5* mutants accumulated significantly higher levels of SA and SAG than WT plants against the *Pto* DC3000. On the other side, the free JA, JA-Leu/Ile, and ABA levels did not differ considerably between *aha5* mutants and WT Col-0 plants post-*Pto* DC3000 infection or buffer infiltration (Figure 6a–e). Besides, the expression levels of genes related to the SA defense signaling pathways were measured in *aha5* mutants and WT before and after the *Pto* infection. Consistent with the changes of defense hormones, gene expressions of *SID2* (SA synthesis-related gene) and *PR1* (SA response gene) were significantly induced in *aha5* mutants after the *Pto* inoculation compared to WT plants (Figure S5). These results demonstrate that the mutation in *AHA5* might enhance SA accumulation and the related defense signaling pathway, contributing to the enhanced plant disease resistance against the *Pto* DC3000 pathogen infection. 

### 2.7. AHA5 Interacts with RIN4 In Vitro and In Vivo

**Figure 7:**
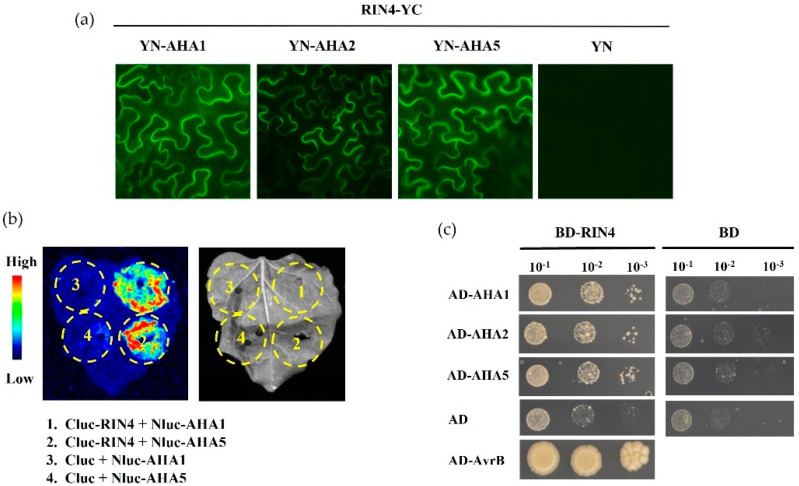
AHA5 interacts with RIN4 in vivo and in vitro. (**a**) Protein interaction between AHA5 and RIN4 by BiFC assay. The full length of AHA1/2/5 was fused with the Nterminus of YFP (YN), while the full length of RIN4 was fused with the C-terminus of YFP (YC). Agrobacterium strains containing the related constructs of AHAs (YN-AHA1, YN-AHA2, and YN-AHA5) and RIN4 (RIN4-YC) were co-infiltrated into *N. benthamiana* leaves. The empty YN construct (YN) was used as the negative control. The signals were checked 3 days after the infiltration of *N. benthamiana* leaves. The yellow fluorescent signals indicate the interaction. (**b**) Protein interaction between AHA5 and RIN4 by luciferase assay. The interaction between AHA1/5 and RIN4 was determined by a split luciferase complementation assay in *N. benthamiana*. Luciferase activities were detected by luminescence imaging with a CCD camera. AHA1/5 and RIN4 were fused with Nluc and Cluc as Nluc-AHA1/5 and Cluc-RIN4. The yellow dotted circles show leaf regions that were infiltrated by the Agrobacterium strains containing the related constructs. The empty Cluc construct (Cluc) was transformed as the negative control. The signals were checked 3 days after the infiltration of *N. benthamiana* leaves. The signals indicated the interactions. Numbers 1–4 show different combinations of constructs labeled on the right. (**c**) AHAs-RIN4 interactions were detected by the yeast two-hybrid assay. RIN4 (full length) and AHAs (C-termini) were fused with the DNA-binding domain (BD) and activation domain (AD) of the GAL4 transcription factor, respectively. BD-RIN4 and AD-AHAs were co-transformed into yeast cells. Empty AD and BD were used as the negative controls. The RIN4–AvrB interaction was used as a positive control. A series of diluted yeast cells were grown on the synthetic dropout media, which lacked Trp, Leu, and His (−Trp−Leu−His) and was supplemented with 3-AT. The growth of the yeast cells could indicate the existence of protein interactions.

RIN4 plays critical functions in PTI and ETI, and it was reported to form a plant immunity signaling hub, recruiting multiple known and unknown components involved in plant defense as the RIN4 protein complex [7]. Since AHA1 was critical in stomatal defense and interacted with RIN4 for the related plant disease resistance [9,11], the interaction experiments were carried out to determine whether AHA5 could interact with and function via RIN4. Firstly, the bimolecular fluorescence complementation (BiFC) assay was conducted to detect the possible interaction between AHA5 and RIN4. AHA1, AHA2, and AHA5 were fused with the N-terminus of yellow fluorescence protein (YFP), while RIN4 was fused to the C-terminus of YFP. To detect complementary fluorescence, individual AHAs and RIN4 were co-infiltrated into *N. benthamiana* leaves. The results showed that AHA5 interacted with RIN4, which is the same as AHA1 and AHA2 (Figure 7a). To further confirm the in vivo protein–protein interactions of RIN4-AHA5, the luciferase complementation imaging (LCI) assay was also carried out to detect the interaction. RIN4 and AHA1/5 were constructed with the C-terminus and N-terminus of luciferase, respectively. RIN4 and AHA1/5 were co-infiltrated into the *N. benthamiana* leaves. The results showed that the signals appeared at the spots of AHA1 and AHA5 infiltration, suggesting strong interactions between RIN4 and AHA1 and AHA5, which was consistent with the results of the BiFC assay (Figure 7b). In addition, the yeast two-hybrid assay detected the physical interactions of proteins. AHA1, AHA2, and AHA5 were fused with the activation domain (AD) of the Gal4 transcription factor, while RIN4 was fused with the DNA-binding domain (BD). The AvrB–RIN4 interaction was taken as the positive control, and the empty vector (pGAD-T7 or pGBK-T7) was used as a negative control. The results showed that all three tested AHA members, including AHA5, could interact with RIN4 in the Y2H assay, suggesting a conserved interaction motif with RIN4 shared between these three AHA members (Figure 7c). Altogether, the results demonstrate the interaction between AHA5 and RIN4 in vivo and in vitro, indicating that AHA5 may be involved in RIN4-mediated plant immunity and function as an important component of the critical versatile docking platform for plant defense [7,8].

## 3. Discussion

Different Arabidopsis PM H^+^-ATPases probably possess divergent roles in plant growth, development, and defense to abiotic and biotic stresses. *AHA1* and *AHA2* together play extensive roles in plant growth and development since the double-knockout lines are lethal [9]. Both *AHA1* and *AHA2* are involved in the blue-light-mediated stomatal movement [49,50]. *AHA1* is also involved in plant defense against bacterial pathogens through affecting the Arabidopsis stomatal movement. The constitutive expression mutant of *AHA1* (*ost2-1D*) exhibited active H^+^-ATPase activity with the wider stomatal apertures than the WT plants, and this mutant showed susceptibility to *Pto* DC3000 by the spray inoculation [9,51]. ABA was known to negatively regulate PM H^+^-ATPase activity [52]. It was found that an endosome trafficking component, VAMP711, regulates the ABA-mediated inhibition of PM H^+^-ATPase activity and stomatal closure to drought stress through interacting with AHA1 and AHA2 [48]. *AHA2* is the most abundant H^+^-ATPase in the primary root. The *aha2* mutant plants exhibited a reduced ability to acidify the surroundings of the roots, which leads to a lower nutrient uptake and the reduced growth of *aha2* mutants [53,54]. AHA2 and AHA7 were studied to regulate the root tip H^+^-efflux in response to low-phosphorus stress. Meanwhile, AHA2 mainly modulates the primary root elongation and AHA7 mainly mediates the root hair formation [23]. 

**Figure 8:**
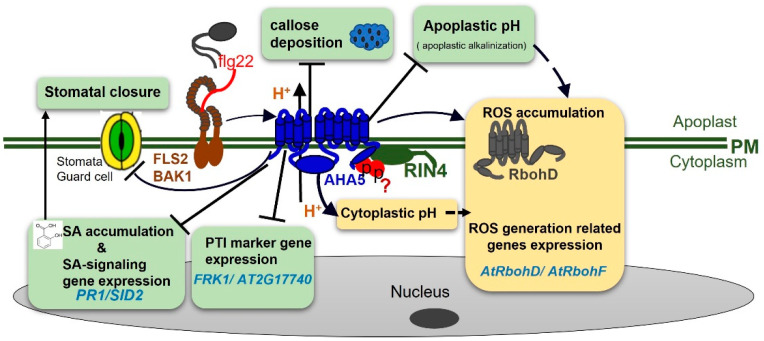
The hypothetical working model of the function of AHA5 involved in plant PTI upon the bacterial *Pto* infection. During the interactions, the bacterial pathogens, such as *Pto* bacteria strains, invade plants via stomata and release PAMPs. The perception of PAMPs (for example, flg22 in red) by plant PRRs (for example, FLS2-BAK1 complex) leads to the activation of the downstream defense components, including RbohD and AHA5. RbohD is responsible for converting O_2_ to O_2_^−^ to further form H_2_O_2_. AHA5 (associated with RIN4) could pump H^+^ out of cytosol to the apoplast, leading to apoplastic acidification. Therefore, the mutation of *AHA5* contributes to the apoplastic alkalinization. Moreover, *AHA5* is positively involved in the production of H_2_O_2_ and ROS synthesis-related gene expression (*AtRbohD* and *AtRbohF*) during PTI. Although the *aha5* mutants exhibited lower levels of H_2_O_2_ and ROS-related gene expression, the mutant plants still conferred the enhanced resistance against the *Pto* infection. The reason is that AHA5 is negatively involved in stomatal closure, callose deposition (blue clot with dots), SA accumulation, SA-signaling-related gene expression (*PR1*/*SID2*), and the other PTI-related gene expression (*FRK1* and *AT2G17740*). All these are critical plant PTI responses upon the pathogen infection. In addition, AHA5 interacts with RIN4, and they possibly function together in plant PTI. The AHA5–RIN4 interaction might be associated with the protein phosphorylation of RIN4 (red circle), which needs to be investigated in the future. Furthermore, AHA5 may also crosstalk with the other plant defense components to contribute to PTI, which also needs to be further investigated. Solid arrows show the mechanisms and pathways currently known in this study. Question marks and dotted arrows indicate the unknown or hypothetical mechanisms and pathways.

The functions of the other AHAs are largely unknown. Here, we identified AHA5, one of the most abundant Arabidopsis H^+^-ATPases in guard cells, as a novel and critical component that is negatively involved in PTI against the *Pto* DC3000. As summarized in Table S1 and in the hypothetical working model shown in Figure 8, we provide evidence that AHA5 negatively participates the PTI via its function related to H^+^-ATPase activities, leading to different defense responses. More specifically, AHA5 functions in PTI by negatively affecting the callose deposition, stomata closure, SA accumulation, and SA pathway activation. In addition, AHA5 may cooperate with RIN4 to function in PTI in Arabidopsis. 

ROS accumulation is one of the earliest defense responses in response to PAMP flg22 treatment. ROS bursts are known to be induced by the PM-localized respiratory burst oxidase homologs (RBOHs). RbohD is the most important RBOH for ROS production during plant innate immunity. RbohD produces superoxide anions (O_2_^−^) by transferring electrons (from NADPH) to oxygen molecules, and the oxygen anions require H^+^ to produce HO_2_ intermediate, which decays into H_2_O_2_ and O_2_ [37,38,39]. Interestingly, our study discovered that AHA5 is positively involved in H_2_O_2_ production in PTI, which might correlate with its H^+^-ATPase activity (H^+^ out-pumping). We found that the *aha5* mutants produced less H_2_O_2_ than WT plants in response to the flg22 treatment, although the *aha5* mutants were more resistant than the WT plants (Figure 2). However, the flg22-induced H_2_O_2_ accumulation was not significantly reduced in *aha5* mutants when treated with the H^+^-ATPase inhibitor vanadate compared to the WT plants (Figure 3c,d). These findings demonstrated that *AHA5*-associated H^+^-ATPase activity may positively affect the PTI-induced H_2_O_2_ production. PM H^+^-ATPases were responsible for pumping the H^+^ out of the plasma membrane to the apoplast. It was shown that the proton transport through PM H^+^-ATPases controls the cytosolic pH homeostasis and apoplastic pH in Arabidopsis [55]. In our study, the impaired function of *AHA5* showed the reduced levels of pH in the cytosol and higher apoplastic fluid pH in response to the *Pto* DC3000 treatment (Figure 2a–c). Taken together, the apoplastic H^+^ may function as a coupling link between the H_2_O_2_ production and the alkalization of the apoplast. This statement was further confirmed by the decreased transcript levels of *AtrbohD* and *AtrbohF* in *aha5* mutants after the *PtoDC3000* pathogen inoculation (Figure 3e). Generally, ROS accumulation in the apoplast is important for plants to defend themselves against the *Pto* pathogen infections [56]. However, the reduced H_2_O_2_ accumulation to the PAMP flg22 treatment in *aha5* mutants might be more related to the apoplastic alkalization upon the pathogen infection caused by the impaired H^+^-ATPase activity of AHA5 (Figure 8; Table S1). 

In addition to the ROS accumulation, Arabidopsis plants elicit the other fast defense responses during the PTI. As shown in the hypothetical model in Figure 8, *AHA5* is negatively involved in the flg22-induced SA accumulation, expressions of genes in the SA-related pathway, and callose deposition. The defense hormone SA is critical for plant defense against the (hemi) biotrophic pathogens, such as *Pto* DC3000 infection [57]. We found that SA and SAG accumulation were significantly increased in *aha5* mutants against the *Pto* DC3000 infection. Consistent with the defense hormone qualification, the transcript levels of genes related to SA biosynthesis (*SID2*) and the SA response pathway (*PR1*) were significantly higher in *aha5* mutants than WT plants (Figure S5). The defense hormone SA was reported to positively affect FLS2-mediated responses, such as callose deposition [57], indicating that the increased SA accumulation in *aha5* mutants might further enhance the callose formation during the PTI. These results suggest that the callose deposition and SA accumulation might be critical in the *AHA5*-involved PTI. 

The *Pto* pathogen is known to internalize into leaves through stomata specifically. Stomata have been well known to play important roles in innate immunity against the *Pto* invasion and some other pathogens [28,29]. Stomatal movement regulation is found to be an important part of AHA5-mediated resistance (Figure 8; Table S1). We firstly showed here that the impaired function of *AHA5* led to the enhanced resistance with more stomatal closure when sprayed with the virulent *Pto* DC3000. Similarly, it was reported that the constitutively active mutants of *AHA1* (*ost2-1D* and *ost2-2D*) plants were more susceptible than WT plants with the constitutively open stomata when sprayed with *Pto* DC3000 [9]. Thus, *AHA1* and *AHA5* might have similar functions in stomatal defense. Coronatine is the critical virulence factor in *Pto* DC3000 that adjusts stomata to re-open after the PTI-induced closure [29]. We found that the ability of stomata to open in response to coronatine was compromised in *aha5* mutant guard cells, indicating that coronatine is also an integral part of the *AHA5*-associated stomatal defense. For our study, the *aha5* mutants still displayed the enhanced resistance when injected with *Pto* DC3000, while the susceptibility of *ost2-1D* and *ost2-2D* did not differ from WT plants when injected with *Pto* DC3000 [9]. These results suggest that defense pathways other than the stomatal defense are also involved in *AHA5*-involved PTI against the *Pto* pathogen infection (Figure 8; Table S1). 

Plant hormones were found to act as critical signals to regulate stomatal development, movement, and function. Generally, the exogenous application of ABA inhibits stomatal opening, which results in less water loss in the plants [58]. Moreover, stomatal closure is an integral part of the SA-regulated innate immune system. The SA-deficient *nahG* transgenic Arabidopsis plants did not close the stomata in response to the bacterial pathogen infection [29]. The previous study also demonstrated that the treatments of the root-associated *Bacillus* strains could cause the stomatal closure to restrict the invasion of the foliar pathogen *Pto* DC3000 by triggering the SA signaling pathway [59]. When we studied the function of *AHA5* in stomatal defense, we found that *aha5* mutant plants responded to SA to a more substantial extent than those of WT Col-0, with more stomatal closure in the mutant plants. Consistent with the defense hormone qualification, *aha5* mutant plants accumulated significantly higher levels of SA than WT against the *Pto* DC3000 inoculation (Figure 5 and Figure 6). Therefore, it is possible that AHA5 functions in the SA-involved defense pathway and the related stomatal closure (Figure 8). Besides, ABA did not appear to play critical roles in the *AHA5*-associated PTI based on the results that ABA levels did not differ between *aha5* mutants and WT after the *Pto* pathogen infection and the *aha5* mutant plants did not respond to ABA in the guard cells. The *aha5* mutant plants showed similar stomatal apertures to WT, which are same as the stomata phenotype of *ost2-1D* and *ost2-2D* in response to the ABA treatment [52]. 

AHA1/2/5 are the most abundant PM H^+^-ATPases in guard cells [19]. It was reported that RIN4 cooperates with AHA1 and AHA2 to function in plant immunity [9]. RIN4 directly interacts with the C-terminal regulatory domain of the PM H^+^-ATPases AHA1 and AHA2, enhances the H^+^-ATPase activity, and then displays the wider stomatal apertures for the susceptibility to the *Pto* pathogen infection [10]. Interestingly, our study found that AHA5 also interacts with RIN4 both in vitro and in vivo (Figure 7). This result indicates that AHA5 might function together with RIN4 and play critical roles in the RIN4-associated immunity platform during PTI (Figure 8; Table S1). Besides, *AHA1* and *AHA2* play critical roles in plant growth and development since double-knockout lines were lethal [9]. Our study showed that two knocked-down mutants of *AHA5* exhibited similar growth and development as WT plants, indicating that *AHA5* might not be critical for plant growth and development but only defense. Whether *AHA5* could function together with *AHA1/2* to be involved in plant growth/development and plant defense needs further investigation.

In conclusion, we firstly identified that *AHA5* is negatively involved in PTI in Arabidopsis, which leads to diverse defense responses by affecting the H^+^ out-pumping, stomatal closure, callose deposition, defense gene expression, SA accumulation, and interaction with RIN4 during PTI. Potentially, *AHA5* may crosstalk with different plant-defense-related components to contribute to PTI. There are still large knowledge gaps on whether and how these related components coordinate with each other in PTI. Further studies of PM H^+^-ATPases in the other plant species could facilitate our understanding on their biological functions in a broader way, which may help us to develop strategies to apply them in improving crop growth and health in agricultural production.

## 4. Materials and Methods

### 4.1. Plant Growth and Pathogen Inoculations

Arabidopsis plants were cultured for the phenotype observation and pathogen inoculation in a growth room with a temperature of 26 °C during daytime and 20 °C at night, 80% humidity, 14 h of light, and 10 h of darkness. The genotyping to screen the homozygous lines of two *aha5* mutants, *aha5-1* (SALK_147597) and *aha5-2* (SALK_127844), was followed the instruction developed by the Salk Institute Genomic Analysis Laboratory (http://signal.salk.edu/tdnaprimers.2.html) [30]. The forward (RP), reverse (LP), and left border primers (LBb1.3) for *aha5* mutant genotyping is listed in Table S2. The confirmed homozygous lines were selected for the further study.

Pathogen inoculations were conducted on the plants 4–6 weeks after transplanting. Bacterial growth assays were performed by infiltrating bacterial broth with a concentration of 10^5^ colony-forming units (CFU)/mL (OD_600_ = 0.0002) in 10 mM MgCl_2_ into the abaxial side of the leaves with a 1 mL syringe. After infiltration, the residues were wiped off the leaves, and the plants were returned to the growth room 30 min later. At 3 days after the infiltration (dpi), the leaf discs were collected into five technical replicates containing three leaf discs each. The bacterial titer in each technical replicate was determined by grinding the leaf discs to homogeneity in 10 mM MgCl_2_ with a serial dilution and plating onto the King’s B plates [60]. Colonies were counted and used to calculate the mean CFU/cm^2^ for each treatment, and the final values were log-transformed. The log-transformed means from individual replicates, as single data points, were then combined from multiple independent biological repeats and used to calculate the mean and standard error. Significant differences were determined either between different bacterial strains in the same plant background or between plants infiltrated with the same bacterial strains. *Pto* DC3000 and *Pto* DC3000 *hrcC−* were cultured on King’s B plates with rifamycin. The bacterial cells were scratched from the plates and suspended with proper solutions depending on the related experiments. When the bacteria were used for injection, the concentration was 10^5^ CFU/mL. When the bacteria were used for spray inoculation, the concentration was 10^9^ CFU/mL. The spray inoculation was performed with a vacuum-aided spray nozzle. The vacuum pressure was set to 25 psi. After the leaves were dry, the plants were put back in a growth chamber and covered to retain moisture. As mentioned above, the bacterial growth was checked at 3 dpi by plating. 

### 4.2. Phylogenetic and Alignment Analysis

The amino acid sequences of the AHAs (11 in total) in the Arabidopsis genome were applied to the alignment and phylogenetic analysis with the MegAlign program in the Lasergene package. The alignment was performed with the method of Clustal W [61]. The phylogenetic tree was constructed based on the alignment with the default parameters and was displayed using the straight branches and cladogram tools. 

### 4.3. qPCR Analysis of Gene Expression

Arabidopsis plants (treated or untreated) were collected as described for RNA extraction (Trizol reagents). Total RNA was extracted using Trizol (Cat #: T9424, Sigma-Aldrich, St. Louis, MO, USA). The RNA concentration of each sample was measured by NanoDrop (Thermo Fisher Scientific, Columbus, OH, USA). The same amount of RNA (1 μg) of each sample was applied for the first-strand cDNA synthesis (Reverse Transcripase, CMV). The qPCR was performed using the 96-well blocks and Universal SYBR^®^ Green Supermix (Bio-Rad, Hercules, CA, USA) with the CFX96 real-time PCR detection system (Bio-Rad, Hercules, CA, USA). The primer pairs used for qPCR in this study are listed in supplemental data Table S2. The relative gene expression of *AHA5* in WT Col-0 and *aha5* mutants by qPCR was normalized to *ACTIN*’s expression. The other gene expressions in WT and *aha5* mutants by qPCR were normalized to *UBIQUITIN*’s expression. The values of the experimental controls (untreated Col-0) were set as one. All the values were relativized to such experimental controls and are displayed as the untreated average fold for Col-0 [62].

### 4.4. The Cytoplasmic pH Gradient Staining 

Arabidopsis leaves of *aha5* mutants and WT at 4 weeks old were syringe infiltrated with *Pto* DC3000 at a concentration of 1 × 10^8^ CFU/mL or MgCl_2_ buffer (10 mM). The inoculated leaves were cut one hour after the inoculation and then submerged in 10 µM BCECF-AM stain solution (Cat #: 14562, Sigma-Aldrich, St. Louis, MO, USA) for 15 min in the dark at room temperature. Then, the leaves were rinsed with water three times (1 min each). After being washed with water, the fluorescent signal of the leaf peel samples was checked under the confocal microscopy (Nikon A1+, Tokyo, Japan). The BCECF-AM dye was excited with a 488 nm laser, and fluorescence was collected at 530 nm [34,35]. The fluorescent signal intensity was determined with ImageJ software.

### 4.5. Apoplastic Fluid Extraction and Apoplastic pH Evaluation

Apoplastic fluid was extracted according to the infiltration-centrifuge protocol method developed by Gentzel et al. [36] with some modifications of the extraction buffer and centrifuge speed and time that are more suitable for Arabidopsis apoplastic fluid extraction (shared and unpublished protocol from Dr. David Mackey’s lab). Briefly, Arabidopsis leaves of 3-week-old mutant and WT Col-0 plants were sprayed with *Pto* DC3000 at a concentration of 1 × 10^9^ CFU/mL or MgCl_2_ (10 mM), respectively. The inoculated leaves with the same development stage were cut and vacuum infiltrated with infiltration fluid containing 10–20% methanal. Through a thorough infiltration, the leaves showed a uniformly darker color and translucent appearance. Then, the surfaces of these infiltrated leaves were dried using tissue paper wipes. The apoplastic fluid was harvested by inserting the leaves into 2 mL tubes (three leaves for each tube) and centrifuging at 6000× *g* for 10 min at 4 °C. All steps were performed at 4 °C, including buffer storage, leaf-cutting, and temporary storage. The supernatant was collected from one treatment after centrifuge. The pH of the apoplastic fluid was measured immediately with a micro-electrode (Horiba LAQUAtwin PH-22 Compact PH meter, #3999960123, Kyoto, Japan). In this study, 50–60 leaves were collected to obtain enough aploplastic fluid for each treatment, in which at least five biological replicates were applied. The related experiments were repeated three times with the consistent results.

### 4.6. H_2_O_2_ Assay

The H_2_O_2_ assay method was described previously [56,63]. Briefly, the Arabidopsis leaf discs were cut (7 mm in diameter) and suspended in water for around 36 h to remove the wounding related H_2_O_2_ production. To further detect the H_2_O_2_ production, two leaf discs were soaked in 100 mL of reaction solution with the luminol substrate (Immuno-Star horseradish peroxidase substrate 170-5040, Bio-Rad, Hercules, CA, USA), 1.0 μL of peroxidase (1 mg/mL), and 1.0 μM of flg22 PAMP. Immediately after the addition of all the components, the luminescence was measured continuously for 1 s at 10 s intervals for 30 min with a Glomax 20/20 single well luminometer (Promega). Three replicates were performed for each treatment. The experiments were repeated three times with consistent results. 

### 4.7. Callose Deposition Assay

A callose deposition assay was conducted as described previously [64]. Briefly, leaves of 4-week-old plants were infiltrated with 10 mM MgCl_2_ or 50 μM flg22 (PAMP). At 16 h after infiltration, leaves were collected, cleared with lactophenol, washed with 50% ethanol, then with water, stained with 0.01% aniline blue (Cat #: 415049, Sigma-Aldrich, St. Louis, MO, USA), dissolved in 150 mM K_2_HPO_4_ (pH 9.5), mounted on slides in 50% sterile glycerol, and examined with a Nikon Eclipse 80i epifluorescent microscope (Nikon, Tokyo, Japan). Five or more individual leaves were applied for the analysis of each treatment. Images were captured from a similar position on each leaf. Then, the numbers of callose depositions were determined using ImageJ software [65].

### 4.8. Stomata Closure Assays

Stomata closure assays were conducted following the method described previously [29]. Briefly, 5–6-week-old plants grown at 22 °C with a 14 h photoperiod were chosen to detect the stomata’s response to different treatments. The *Pto* DC3000 suspension (10^8^ CFU/mL), the phytohormones (SA and ABA), and coronatine were prepared to final concentrations of 500 μM, 50 μM, and 1 mg/mL in the MES buffer (25 mM MES-KOH (pH 6.15) and 10 mM KCl). Before the treatments with *Pto* DC3000 and phytohormones (SA/ABA), which were supposed to trigger stomatal closure, the Arabidopsis plants were exposed to light for 2 h to induce stomatal opening. On the other side, coronatine was supposed to trigger stomatal opening. The Arabidopsis plants were kept in the dark for 2 h before the coronatine treatment. After preparation, the fully expanded leaves were excised from the plants and incubated in the MES buffer of different treatments with the abaxial epidermis contact solution. Pure MES buffer was used as a mock control. For the *Pto* DC3000 treatment, the abaxial epidermis was peeled off to check the stomata responsiveness with the Nikon Eclipse 80i epifluorescent microscope at 0 h, 2 h, and 4 h post-incubation. In addition, the stomata aperture was checked with the microscope at 0 h and 4 h post-incubation for the phytohormones and coronatine treatments [10,66,67,68]. 

### 4.9. Phytohormone Quantification

Arabidopsis leaves were injected with the *Pto* DC3000 or MgCl_2_. Leaves of 120 mg fresh weight were harvested 48 h after the injection, put in liquid nitrogen, and stored at −80 °C [69]. The tissues were ground and extracted with the extraction buffer (10% methanol and 1% acetic acid in water) by following the method described previously [61]. Isotope-labeled internal standards were added to the tube at the beginning of the extraction. The amounts of internal standards added were 1 ng of ^2^H_6_ ABA (d_6_-ABA, Toronto Research Chemicals, North York, ON, USA, part #: A110002), 10 ng of ^2^H_5_-JA (d_5_-JA, CDN Isotopes, Pointe-Claire, QC, Canada, part #: D-6936), and 15 ng ^2^H_6_-SA (d_6_-SA, CDN Isotopes, Pointe-Claire, QC, Canada, part #: D-1156). Extraction controls were set for each extraction with no plant material added. For accurate extraction, the plant leaf tissues were extracted three times with 400 μL of extraction buffer for each time. After adding the extraction buffer, the tubes were set on ice for 30 min each time. The extracts were centrifuged, and the supernatants were pooled for each sample separately. Eventually, the samples were applied to the UPLC/ESI/MS analysis with the Thermal Fisher Ultimate 3000 system (Thermal Fisher, Columbus, OH, USA). The UPLC separation was carried out on a Waters 3 μm C18 (100 mm × 2.0 mm) column at 35 °C. The mobile phase was set for a continuum gradient from (94.9% H_2_O: 5% CH_3_CN: 0.1% CHOOH) to (5% H_2_O: 94.9% CH_3_CN: 0.1% CHOOH) over 20 min. The analysis of the compounds was based on multiple reaction monitoring (MRM) of ion pairs for the labeled and endogenous hormones. The transition settings for SA, JA, and ABA were ^2^H_6_-SA 141 (97), SA 137 (93), ^2^H_6_-ABA 269 (159), ABA 263 (153), SAG 299 (93), ^2^H_5_-JA 211 (61), and JA 209 (59). The daughter masses were denoted in the brackets listed above as reported previously [61]. 

### 4.10. Bimolecular Fluorescence Complementation (BiFC) Assay

Full-length RIN4 and AHA 1/2/5 proteins were fused with the N-terminus (pDEST-^GW^VYNE vector) and C-terminus (pDEST-^GW^VYCE vector) of yellow fluorescence protein (YFP) with the GATEWAY cloning vector system. Empty vectors were used as the negative controls. The constructs were introduced into Agrobacteria stain GV3101, which were then used for Agro-infiltration to detect the potential interaction of these two proteins in tobacco (*Nicotiana banthiamiana*) leaves. The Agrobacteria harboring either N-terminus or C-terminus fusion constructs were cultured to OD_600_ = 0.8 and then concentrated by being centrifuged and suspended in the injection solution (10 mM MgCl_2_, 10 mM MES, pH 6.5). A proper combination of the *Agrobacteria* was mixed for injection. The concentration for each construct in the *Agrobacteria* mixture was OD_600_ = 1.0. Tobacco plants were used to perform the infiltration 4–6 weeks after transplanting. The infiltrated leaves of 3 dpi were observed for fluorescence signals (interactions) with the Nikon Eclipse 80i epifluorescent microscope (Nikon, Tokyo, Japan) [70].

### 4.11. Luciferase Complementation Imaging (LCI) Assay

Full-length AHA1 or AHA5 and RIN4 were fused with the N-terminus and C-terminus of luciferase, respectively. Empty vectors were used as the negative controls. The constructs were introduced into *Agrobacteria* strain GV3101. The NLuc-AHA1/5 Agrobacteria and CLuc-RIN4 *Agrobacteria* were cultured and co-infiltrated into the leaves of *N. benthamiana* in equal amounts (OD_600_ = 0.8 each). The leaves were checked for the potential interactions of target proteins at 3 dpi. The leaves were sprayed with 1 mM luciferin before imaging. The imaging was carried out with a Bio-Rad ChemiDoc XRS+ system (Bio-Rad, Hercules, CA, USA). The signals were collected for 10 min [71]. 

### 4.12. Yeast Two-Hybrid (Y2H) Assay

The N-termni of AHA1/2/5 and full-length RIN4 were fused with the activation domain (AD) and DNA-binding domain (BD) of the GAL4 transcription factor, respectively. Empty vectors were used as the negative controls. Plasmids containing AHAs and RIN4 were co-transformed into yeast cells to detect the possible interactions. The transformation of yeast cells was described previously with modifications [72]. Briefly, yeast (strain MaV203) competent cells were prepared with 100 mM LiAc induction for 30 min at 30 °C. After induction, the competent cells were suspended with the suspension solution (30% PEG3350, 100 mM LiAc, 250 ng/mL Salmon DNA). One microgram of each plasmid DNA was mixed with 200 mL of competent cells. Then, the mixture was incubated for 30 min at 30 °C. Following the incubation, the competent cells were heat-shocked at 42 °C for 30 min (inverting the tubes every 5 min). After heat shock, the transformation of yeast cells was achieved. The yeast cells were applied to the selection medium (SC/-Leu-Trp). Only the co-transformants could grow on the selection medium in 2–3 days at 30 °C. After that, the co-transformants were cultured on the detection medium (SC/-Leu-Trp- His, with 3-AT) to detect the interactions. 

## Data Availability

The data used to support the findings of this study are available from the corresponding author upon request.

## Supplementary Materials

The following supporting information can be downloaded at: https://www.mdpi.com/article/10.3390/ijms23073857/s1.

## Abbreviations

BCECF-AM	[2′, 7′-Bis (2-carboxyethyl)-5 (6)-carboxyfluorescein tetrakis (acetoxymethyl) ester.
PM H^+^-ATPases	plasma membrane H^+^ pumps
