# Supplementary material for: Arabidopsis Plasma Membrane ATPase AHA5 Is Negatively Involved in PAMP-Triggered Immunity
Source: Int J Mol Sci. 2022 Mar 31;23(7):3857. doi: 10.3390/ijms23073857 (PMC8998810; doi:10.3390/ijms23073857)
Supplement: Supplementary file 1 [file ijms-23-03857-s001.zip › ijms-1644469-supplementary.pdf]

International Journal of *Molecular Sciences*

MDPI

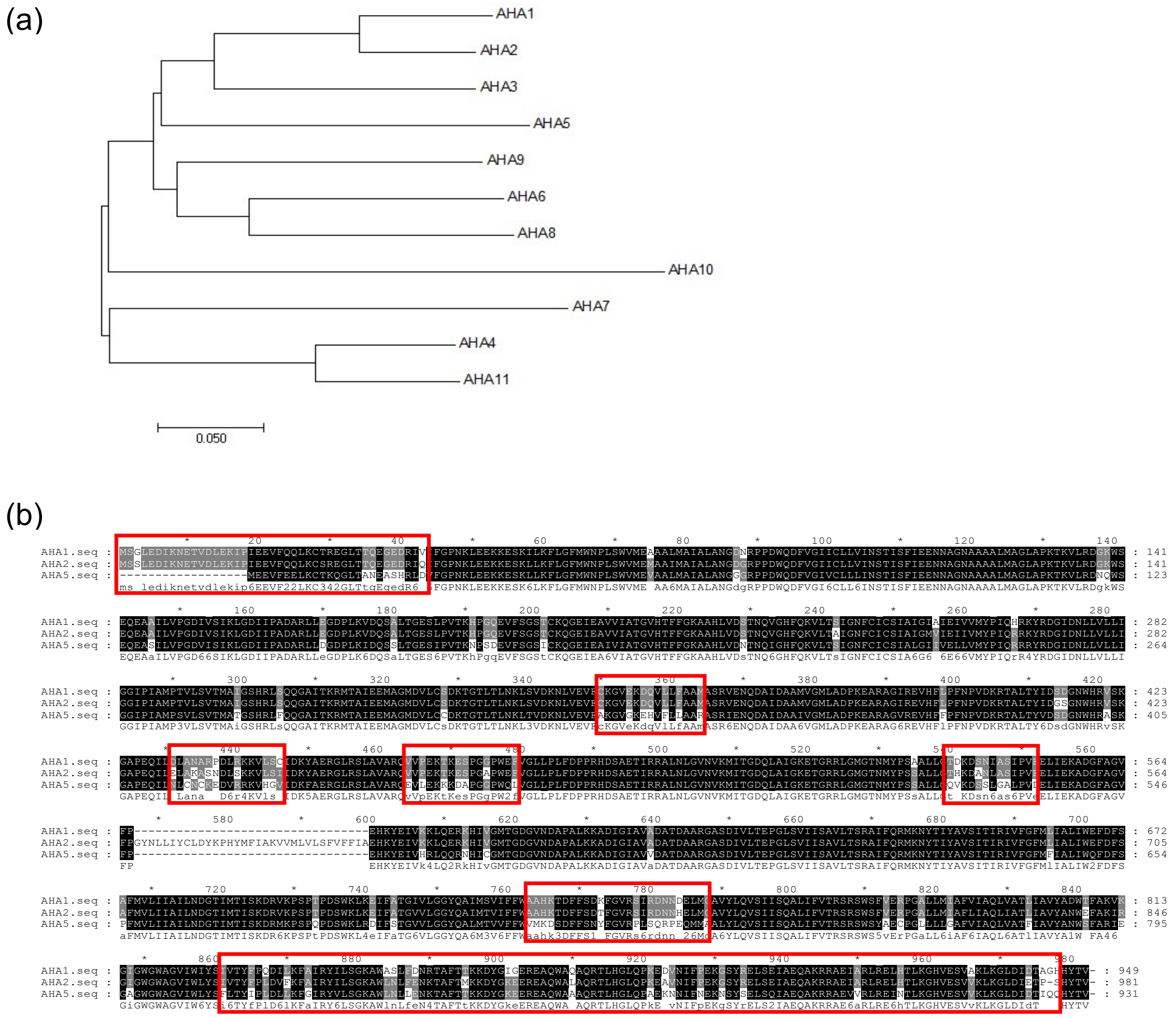

Figure S1. AHA5 differentiates from AHA1 and AHA2. (**a**) Arabidopsis genome harbors 11 AHAs. The Phylogenetic tree of the AHAs family was constructed by the phylogenetic analysis with the MegAlign program in Lasergene package. (**b**) Multiple alignment of amino acids sequences of AHA1, AHA2, and AHA5 was carried out with the MegAlign program in Lasergene package. Black shades indicate the identical sequences. Substitutions are in white. Consensus sequence is shown underneath the aligned sequences. Red boxes highlight 7 main variable regions of AHA5 compared to AHA1/2.

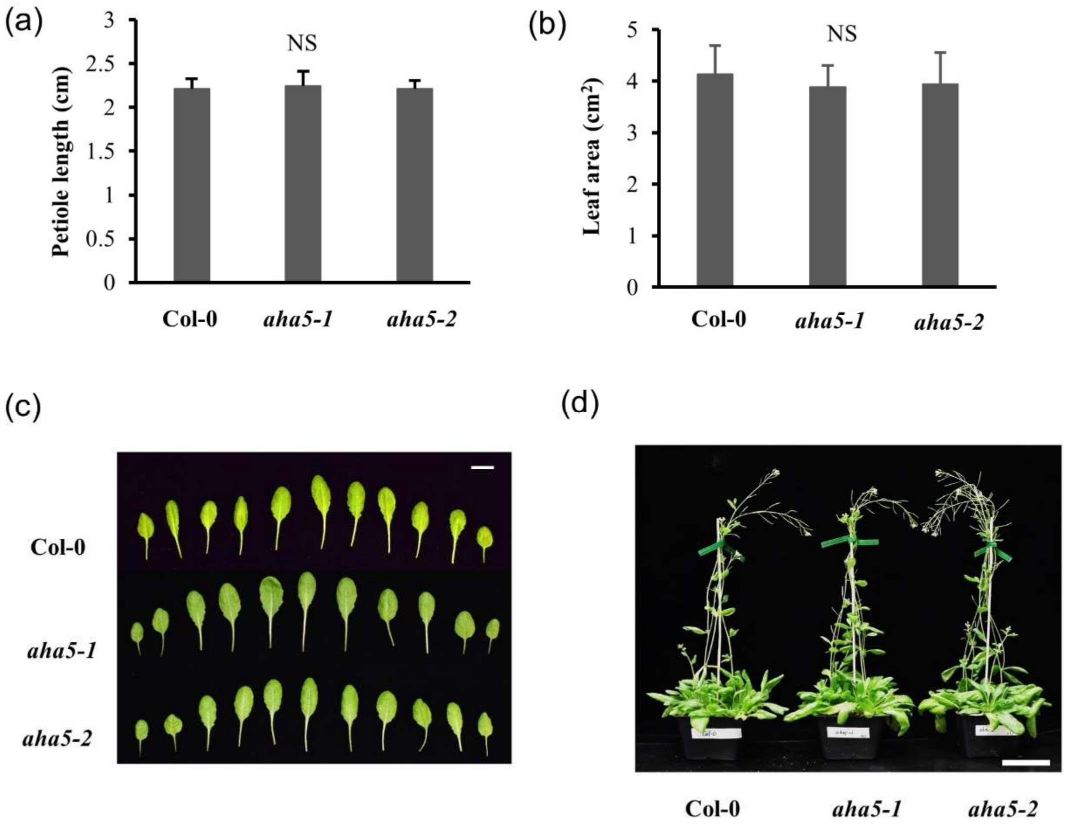

Figure S2. Morphology characterization of the *aha5* mutants and WT Col-0 plants. The *aha5* mutants showed no obvious growth defects compared to WT Col-0 plants. (**a**). Petiole length of the 4-week-old *aha5* mutants and WT plants. (**b**) Average leaf areas from a similar developmental stage of the *aha5* mutants and WT plants. Data represent means ± SE (n = 3), which are from one of the three independent repeats with consistent results. Significant differences between the *aha5* mutants and WT plants were indicated by the asterisks determined from the student's *t*-test ($p < 0.05$). NS represents no significance. (**c**) Leaf phenotypes of rosettes from 4-week-old *aha5* mutants and WT plants. Scale bar indicates 2 cm. (**d**). Morphological phenotypes of the 6-week-old *aha5* mutants and WT plants. Scale bar indicates 6 cm.

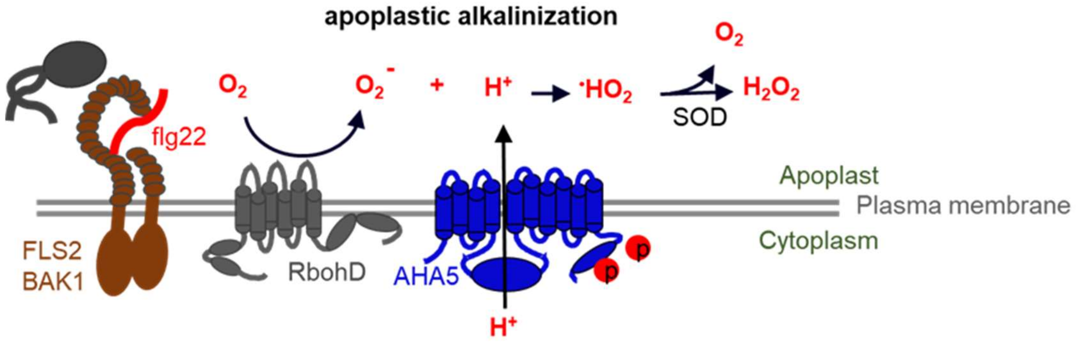

Figure S3. Summary of the involvement of AHA5 in the PTI-induced H_2O_2 production. ROS is generated by RbohD, which converts O_2 to O_2^- by transferring electrons from NADPH to O_2 molecules. The O_2^- and apoplastic H^+ spontaneously forms intermediate $^{\bullet}HO_2$, which is converted to H_2O_2 and O_2 by superoxide dismutase (SOD). The production of ROS (mainly H_2O_2) depletes the apoplastic H^+, which leads to the alkalization of the apoplast.

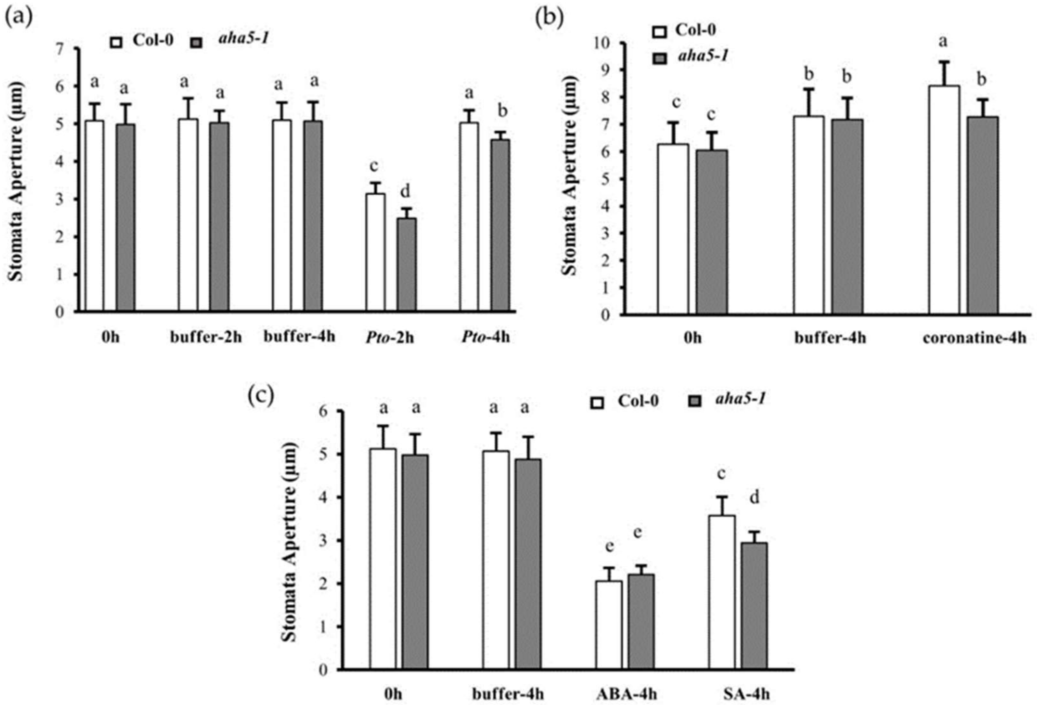

Figure S4. *AHA5* is involved in the stomatal movement upon the pathogen and hormone treatments. (**a**) The *aha5-1* mutant showed the enhanced stomatal closure against the *Pto* pathogen. At the six-week-old stage, the *aha5-1* mutant and WT Col-0 leaves were treated with the cell suspension (10^8 CFU/ml) of the virulent strain *Pto* DC3000. The stomatal apertures were checked with the microscopy at 0h, 2h, and 4h after the pathogen infection with the abaxial epidermis peels. (**b**) The *aha5-1* mutant plants did not respond to the coronatine treatment as the WT plants. The *aha5-1* mutant and WT Col-0 leaves at the six-week-old stage were placed in the buffer or buffer containing 1ng/μl coronatine. Stomatal apertures were checked with the microscopy at 0h and 4h after the treatments. (**c**) The *aha5-1* mutant plants displayed SA-induced stomatal closure compared to the WT plants. The *aha5-1* mutant and WT Col-0 leaves at the six-week-old stage were treated with 50μM ABA or 500μM SA or MES buffer. The stomatal apertures were checked at 0h and 4h after the treatments. All the experiments related to stomata regulation with different treatments were repeated three times with similar results. Data represent means ± SE (n = 50), which are from one of the three independent repeats. Different letters a-e within the figure (**a-c**) indicate the significant differences at $p < 0.05$, which was calculated by one-way analysis of variance (ANOVA) using SPSS ver. 21. (IBM SPSS Statistics, New York, U.S.).

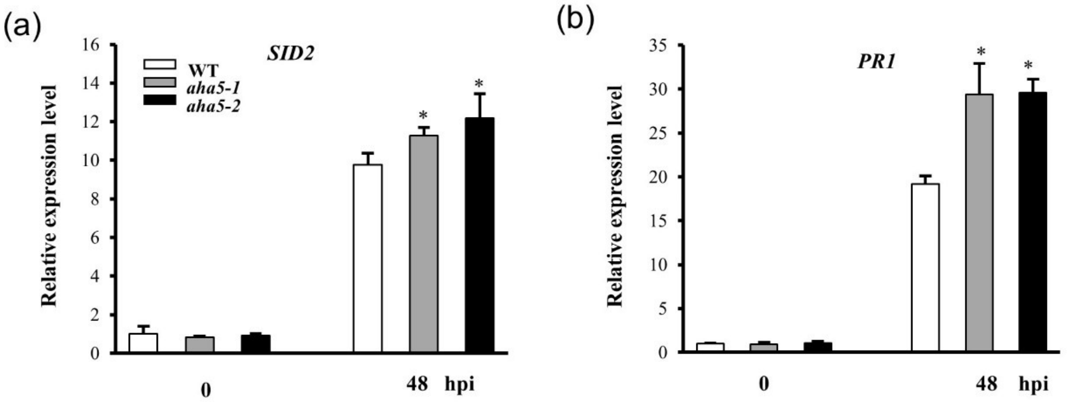

Figure S5. Expressions of SA pathway-related genes in *aha5* mutants and WT plant leaves with the *Pto* DC3000 inoculation at 0 and 48hours thereafter. The gene expressions of *SID2* (SA synthesis-related gene) (**a**) and *PR1* (SA response gene) (**b**) in 4-week-old *aha5* mutants and WT plant leaves were quantified by qPCR and normalized to the expression of *UBIQUITIN*. Data represent means ± SE (n = 4), which are from one of the three independent repeats with consistent results. Significant differences between the WT and *aha5* mutants were indicated by the asterisks determined from the student's *t*-test (* $p < 0.05$).

Table S1. The specific functional roles of AHA5 involved in in plant PTI upon the bacterial Pto infections.

Functional roles of AHA5 in PTI	Detail responses during PTI	Evidence in this study
Negative	The *aha5* mutants displayed the enhanced resistance against the *Pto* pathogens.	Figure 1.
	PTI marker gene expression (*FRK1*/ *AT2G17740*) increased in *aha5* mutants upon *Pto* DC3000 infection.	Figure 1.
	Apoplastic pH increased in *aha5* mutants upon *Pto* DC3000 infection.	Figure 2.
	Callose deposition enhanced in *aha5* mutants upon *Pto* DC3000 infection.	Figure 4.
	Stomatal closure induced in *aha5* mutants upon *Pto* DC3000 infection.	Figure 5.
	SA accumulation enhanced in *aha5* mutants upon *Pto* DC3000 infection.	Figure 6.
	SA pathway related gene expression (*PR1*/*SID2*) increased in *aha5* mutants upon *Pto* DC3000 infection.	Figure S4.
Positive	ROS accumulation decreased in *aha5* mutants in response to PAMP treatment.	Figure 3.
	ROS generation related genes expression (*AtRbohD*/ *AtRbohF*) decreased in *aha5* mutants upon *Pto* DC3000 infection.	Figure 3.
	Cytoplastic pH decreased in *aha5* mutants upon *Pto* DC3000 infection.	Figure 2.
	AHA5 interacts with RIN4 in vivo and in vitro, which might be involved in PTI.	Figure 7.

Table S2. The primer list for the PCR and RT-qPCR.

Name	Sequence 5' to 3'
aha5-1-LP (F1)	GTTCATAGGCTGCAACAAAGG
aha5-1-RP (R1)	GTAGGTGCATACCAGCTGAGC
aha5-2-LP (F2)	TAACACTCAATCCGGGTTCTG
aha5-2-RP (R2)	AAGGAGCTCCAGAGCAGGTAC
LBb1.3	ATTTTGCCGATTTCGGAAC
AHA1-qF	GCTATGGCTTCTAGGGTGG
AHA1-qR	GCCAGTTACCATCAGAGTCG
AHA2-qF	TGAACGTCCTGGAGCATTG
AHA2-qR	TTCCCAGTTGGCGTAAACC
AHA5-qF	GAAGAAAGATGCTCCTGGTG
AHA5-qR	CCTGATTGTCTCGGCACTGT
SID2-qF	CACGGAGTGTCCCACTTCG
SID2-qR	CGTCATGTCATCAGCGGTATC
PR1-qF	GGCTCATATACCTCTGCACTCTA
PR1-qR	TGGTTTAGATACTCTGCTACGGC
PDF1.2-qF	AATAGGAATTGATCCAGTCGCAG
PDF1.2-qR	CTTTCGTCGCCTTACACTCTTT
ACTIN-qF	ATCCAATCCTCCCCAACACC
ACTIN-qR	AACTCTGTCCTTTCTCTTCTCC
UBIQUITIN-qF	AGATCCAGGACAAGGAGGTATTC
UBIQUITIN-qR	CGCAGGACCAAGTGAAGAGTAG
MYC2-qF	GATGAGGAGGTGACGGATACGGAA
MYC2-qR	CGCTTTACCAGCTAATCCCGCA
AtRbohD-qF	AGCTTCACAATTATTGC ACGAG

AtRbohD -qR	TCTCCAGTTAGGTTTA GCGAAG
AtRbohF-qF	TATTGGAGACCATCTTG CTTGT
AtRbohF -qR	CGTTAAAACCGGTTA GTCGATC
FRK1-qF	ACGGGCATAGTTCCACAAAG
FRK1-qR	CGTCAAAAGAACGACGATGA
AT2G17740-qF	CATGCGTTGCTGAAGAAGAGG
AT2G17740-qR	TGCTCCATCTCTCTTTGTGCC
